# The use of randomisation-based efficacy estimators in non-inferiority trials

**DOI:** 10.1186/s13063-017-1837-3

**Published:** 2017-03-09

**Authors:** David Gillespie, Daniel Farewell, Peter Barrett-Lee, Angela Casbard, Anthony Barney Hawthorne, Chris Hurt, Nick Murray, Chris Probert, Rachel Stenson, Kerenza Hood

**Affiliations:** 1 South East Wales Trials Unit, Centre for Trials Research, College of Biomedical and Life Sciences, Cardiff University, Cardiff, UK; 2 Division of Population Medicine, School of Medicine, College of Biomedical and Life Sciences Cardiff University, Cardiff, UK; 3 Velindre Cancer Centre, Velindre Rd., Whitchurch, Cardiff, UK; 4 Wales Cancer Trials Unit, Centre for Trials Research, College of Biomedical and Life Sciences, Cardiff University, Cardiff, UK; 5 Department of Medicine, University Hospital of Wales, Cardiff and Vale NHS Trust, Cardiff, UK; 6 North Adelaide Oncology, Kimberley House, Calvary North Adelaide Hospital, 89 Strangways Terrace, North Adelaide, SA Australia; 7 Gastroenterology Research Unit, Department of Cellular and Molecular Physiology, Institute of Translational Medicine, University of Liverpool, Ashton Street, Liverpool, UK; 8 Division of Infection and Immunity Research, School of Medicine, College of Biomedical and Life Sciences, Cardiff University, Cardiff, UK; 9 Centre for Trials Research, College of Biomedical and Life Sciences, Cardiff University, Cardiff, UK

**Keywords:** Treatment non-adherence, Non-inferiority, Efficacy, Intention-to-treat, Per-protocol, Structural mean models

## Abstract

**Background:**

In a non-inferiority (NI) trial, analysis based on the intention-to-treat (ITT) principle is anti-conservative, so current guidelines recommend analysing on a per-protocol (PP) population in addition. However, PP analysis relies on the often implausible assumption of no confounders. Randomisation-based efficacy estimators (RBEEs) allow for treatment non-adherence while maintaining a comparison of randomised groups. Fischer et al. have developed an approach for estimating RBEEs in randomised trials with two active treatments, a common feature of NI trials. The aim of this paper was to demonstrate the use of RBEEs in NI trials using this approach, and to appraise the feasibility of these estimators as the primary analysis in NI trials.

**Methods:**

Two NI trials were used. One comparing two different dosing regimens for the maintenance of remission in people with ulcerative colitis (CODA), and the other comparing an orally administered treatment to an intravenously administered treatment in preventing skeletal-related events in patients with bone metastases from breast cancer (ZICE). Variables that predicted adherence in each of the trial arms, and were also independent of outcome, were sought in each of the studies. Structural mean models (SMMs) were fitted that conditioned on these variables, and the point estimates and confidence intervals compared to that found in the corresponding ITT and PP analyses.

**Results:**

In the CODA study, no variables were found that differentially predicted treatment adherence while remaining independent of outcome. The SMM, using standard methodology, moved the point estimate closer to 0 (no difference between arms) compared to the ITT and PP analyses, but the confidence interval was still within the NI margin, indicating that the conclusions drawn would remain the same. In the ZICE study, cognitive functioning as measured by the corresponding domain of the QLQ-C30, and use of chemotherapy at baseline were both differentially associated with adherence while remaining independent of outcome. However, while the SMM again moved the point estimate closer to 0, the confidence interval was wide, overlapping with any NI margin that could be justified.

**Conclusion:**

Deriving RBEEs in NI trials with two active treatments can provide a randomisation-respecting estimate of treatment efficacy that accounts for treatment adherence, is straightforward to implement, but requires thorough planning during the design stage of the study to ensure that strong baseline predictors of treatment are captured. Extension of the approach to handle nonlinear outcome variables is also required.

**Trial registration:**

The CODA study: ClinicalTrials.gov, identifier: NCT00708656. Registered on 8 April 2008. The ZICE study trial: ClinicalTrials.gov, identifier: NCT00326820. Registered on 16 May 2006.

**Electronic supplementary material:**

The online version of this article (doi:10.1186/s13063-017-1837-3) contains supplementary material, which is available to authorized users.

## Background

In the majority of randomised controlled trials (RCTs), the primary goal is to investigate the superiority of one treatment over another [1]. However, in some instances, it can be sufficient to demonstrate that a treatment is no worse than another on some outcome of interest. This is particularly true where a standard treatment is already in place (a so-called ‘active control’), and the new treatment could offer substantial benefits on non-primary outcomes such as reduce side effects, reduced costs, simpler dosing regimen, etc. This is the purpose of a non-inferiority (NI) trial, where the aim is to demonstrate that a new treatment is no worse than a standard treatment by more than an acceptable amount [2].

The ‘gold standard’ approach to analysis in a superiority trial is based on the intention-to-treat (ITT) principle, where participants are analysed in the groups to which they were originally randomised [3]. This approach is favoured as it preserves randomisation and, in the case of departures from randomised treatment, makes treatment groups appear more similar; therefore, producing a conservative estimate of treatment effect. However, in a NI trial it is desirable for treatment groups to be as similar as possible, and therefore an ITT analysis is viewed as anti-conservative in this situation [4, 5]. Current recommendations are that a per-protocol (PP) analysis should be conducted alongside an ITT analysis for NI trials [6]. A PP analysis excludes participants with departures from randomised treatment, but assumes that the group of participants who are excluded are similar to those who are included on both observed and unobserved variables; an assumption that is usually deemed implausible [7]. The ideal analytical method would be based on participants who received the treatment to which they were allocated, while maintaining a comparison of groups as randomised (and thus not prone to the selection biases that are common with a PP analysis).

Randomisation-based efficacy estimators (RBEEs), such as Structural Mean Models (SMMs), compare the effect of treatment in the group of participants who were allocated to and adhered to treatment with the group allocated to receive control (or standard treatment) but who *would have* adhered to treatment (had they been allocated to the treatment group) [8]. The approach allows for treatment non-adherence [9] while maintaining a comparison of randomised groups. Fischer et al. have developed an approach for estimating treatment efficacy in randomised trials with two active treatments, a common feature of NI trials [10].

The aim of this paper is to demonstrate the use of RBEEs in NI trials using the methods outlined by Fischer et al., and to appraise the feasibility of these estimators as the primary analysis in NI trials. A brief introduction to randomisation-based efficacy estimators will be given in ‘Methods section’, specifically where the estimators are used in trials with two active interventions. This section will also highlight general steps to fitting these models using standard statistical software, before concluding with a description of the studies used as examples in this paper. ‘Results section’ will present worked examples using data from the studies described in ‘Methods section’, while ‘Discussion section’ will summarise the work of the previous sections and highlight the implications of using these methods in practice.

## Methods

### Traditional approaches to deriving efficacy in RCTs

An ITT analysis is used to determine treatment effectiveness in RCTs [11, 12]. Under certain circumstances (e.g. all participants receive all of the treatment to which they were randomised), an ITT analysis can also be used to estimate treatment efficacy. However, in the presence of non-adherence, or departures from randomised treatment, the most common approach to assessing treatment efficacy in an RCT is to conduct a PP analysis. This analysis excludes participants who are determined to have not adhered to their randomised treatment. However, it fails to maintain a comparison of groups as randomised, and is therefore prone to selection bias [11]. While selection bias is thought to be minimised in trials with blinding, and modified definitions of these populations that adjust for observed confounders can be used, selection bias can never be completely discounted from any analyses that make postrandomisation exclusions or manipulations.

### Structural Mean Models to derive randomisation-based efficacy estimators

By recognising that at the beginning of a trial all participants have two potential outcomes – one if they are treated and one if they are not, a SMM relates a treated participant’s observed outcome to their (potentially counterfactual) outcome that would have been observed had they received no treatment [13]. Standard approaches to fitting a SMM rely on using observed exposure, treating randomisation as an instrument (i.e. assuming that it is independent of both observed and unobserved confounders and only effects outcome through its effect on exposure), and finding a value of the treatment effect such that balance is achieved between groups on the outcome in participants who were not treated [14].

**Fig. 1:**
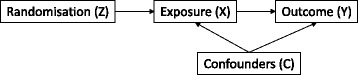
Causal Directed Acyclic Graph (DAG) illustrating using randomisation as an instrument to derive a randomisation-based efficacy estimate

By doing this it becomes possible to derive an estimate of treatment efficacy (the effect that *receiving* treatment has on outcome) that is not prone to the usual selection biases usually found in traditional methods (Fig. 1).

#### SMMs with two active treatments

Conventional SMM methodology is based on trials comparing an active treatment to no treatment (or a placebo). However, in non-inferiority trials it is common to just compare two active treatments – one experimental and one standard. This complicates matters, as without a no-treatment group there is no observed outcome on which to base the potential outcome in the untreated, and therefore the method described above cannot be readily applied.

By identifying baseline covariates that are differentially associated with treatment adherence for each of the treatments, the methodology developed by Fischer et al. allows for the estimation of two distinct causal parameters, from which a contrast can then be made. Identifying baseline covariates that are differentially associated with treatment adherence for each of the treatments, but independent of outcome, allows separate sets of instruments to be derived for each treatment, and allows a potential treatment-free response to be estimated [10].

If suitable baseline covariates are not identified, two distinct causal parameters cannot be estimated. Despite this, a linear contrast can still be made and the following approaches can be taken:

- Fix adherence levels as the same in both arms, and estimate the treatment efficacy in the subpopulation that would always adhere to their treatment at that given level
- Perform sensitivity analyses that vary adherence parameters to explore the impact that differential adherence levels has on outcomes
- Use standard SMM methods and consider the standard treatment as the ‘placebo’ group. This will allow for the comparison of average outcomes at varying levels of the experimental treatment to the average outcome if assigned to the standard treatment (regardless of adherence levels to that standard treatment)

### Example studies

Two non-inferiority trials, whose data were available to the authors, were used to illustrate the proposed methods and its uses and limitations. Beyond the availability of data, the two studies described below were chosen as they were both two-arm non-inferiority trials, with two active treatments involving patients with long-term conditions whose medication use was monitored throughout the trial. The trials differ in terms of the nature of the interventions being compared, with Colitis Once Daily Asacol (CODA) comparing the same treatment prescribed with different regimens, and Zoledronate versus Ibandronate Comparative Evaluation (ZICE) comparing two different treatments with different modes of administration. These examples, while contrasting, are typical of the types of non-inferiority trials conducted and will, therefore, provide useful insight into the methods proposed.

#### The Colitis Once Daily Asacol (CODA) trial

The CODA trial was designed to assess the efficacy and safety of once daily dosing (OD) versus three times daily dosing (TDS) of mesalazine over a 12-month period for patients in remission with ulcerative colitis. The study concluded that the OD regimen was no worse than (non-inferior to) the TDS regimen in terms of clinical relapse using both an ITT and a PP analysis [15]. Research nurses counted the number of tablets returned at each study visit, and deducting this from the number of tablets issued determined the number consumed during the study period. Adherence to study medication in the original trial was defined as participants consuming at least 75% of their issued medication. A subset of participants also had their medication adherence recorded using the Medication Event Monitoring System (MEMS), an electronic monitor that records the date and time of each bottle cap opening. This substudy demonstrated that adherence to study medication was generally lower and more varied for participants allocated to the TDS regimen. However, as this type of measure was not used for all trial participants, it will not be considered further in this paper [16].

#### The Zoledronate versus Ibandronate Comparative Evaluation (ZICE) trial

The ZICE trial was designed to assess whether orally administered ibandronic acid (OIA) was non-inferior to intravenously administered zoledronic acid (IZA) in preventing skeletal-related events (SREs) in patients with bone metastases from breast cancer. The study concluded that orally administered ibandronic acid was inferior to intravenously administered zoledronic acid in both ITT and PP populations [17].

Adherence to study medication was noted by the treating clinician at interim and 12-weekly visits. Participants were defined as having adhered to their allocated treatment if the clinician recorded that study medication had been administered as prescribed during all scheduled visits. See Additional file 1 for more detail.

### Statistical methods

#### Outcomes

For the CODA trial, the outcome of interest was the proportion of participants relapsing during the 12-month study period. The OD regimen was considered to be non-inferior to the TDS regimen as long as the lower bound of the 95% confidence interval of the difference in the proportion of participants in each arm relapsing (OD minus TDS) did not include −0.1.

For the ZICE trial, the outcome of interest for this paper was the proportion of participants experiencing a SRE during the first 12 months of the study. This is a simplified version of the primary outcome from the main paper (time and frequency of SREs), and used for illustration purposes only. There was, therefore, no prespecified non-inferiority margin for this outcome.

#### Modelling approach

##### Determining baseline covariates that differentially predict adherence

Deriving distinct causal estimators for each treatment arm relied on identifying baseline variables that predicted adherence to treatment differently in each arm, while not predicting clinical outcome. Determining these predictors involved two main steps. First, multivariable logistic regression was used to determine the factors that predicted clinical outcome. Variables that were identified univariably at the 20% significance level were entered into the multivariable model, with backward selection used to retain variables independently associated at the 10% significance level. Following this, multivariable logistic regression was used, with the binary adherence variable as the outcome. Predictors of adherence were entered one-by-one into a regression model that included trial arm, and interaction between candidate predictor and trial arm, and the predictors of clinical outcome that were identified during the previous step. Any variables that were associated with adherence at the 20% significance level, as either a main effect or as an interaction with trial arm, were retained in the multivariable regression model. Predictors that remained associated at the 10% significance level were then retained in the final regression model.

For the CODA trial, the candidate baseline predictors used in the outcome and adherence models were age (<65, ≥65 years), age at diagnosis (≤25, 26–45, 46–64, ≥65 years), gender, length of remission (<12 months, ≥12 months), calprotectin concentration (<60 mg/kg stool, ≥60 mg/kg stool), smoking status (never smoker, current smoker, ex-smoker), employment status (unemployed, employed), maximum documented extent of colitis (extensive, left-sided or sigmoid, proctitis), disease duration (≤10 years, 11 to 20 years, >20 years), number of relapses during the past 2 years (1, 2, 3, ≥4), and endoscopy findings (normal, not normal).

For the ZICE trial, the predictors were age, gender, Body Mass Index (BMI), the modified Brief Pain Inventory severity score, quality of life (EORTC QLQ-C30 score version 3.0), SRE within the previous 3 months, previous use of bisphosphonates, treatments being received (including painkilling drugs, chemotherapy, hormone therapy, and trastuzumab).

Variables that were included in the models were checked for notable deviations from linearity. While the relationship between age and outcome in the CODA trial was considered non-linear, this was not the case for the ZICE trial. A cut-off of 65 years was chosen to distinguish between elderly/non-elderly participants (see http://www.who.int/healthinfo/survey/ageingdefnolder/en/).

##### Fitting the structural mean model

The SMM models were fitted using a two-stage, least squares, instrumental variables regression approach. Using this procedure, the trial arm (the instrument), predictors of outcome, and differential predictors of adherence were used to estimate values of the adherence variables in the first stage. These values were then regressed onto the outcome in the second stage. These regressions were fitted simultaneously in order to avoid standard errors that were artificially large. The Huber-White robust standard error, with additional correction for small samples, was used in order to make correct inferences about the differences in proportions [18]. Table 1 provides sample syntax using Stata (v13.0).

**Table 1:** Sample Stata (v13.0) syntax of the structural mean models described in ‘Methods section’ and fitted in ‘Results section’

The Colitis Once Daily Asacol (CODA) trial
ivregress 2sls < <Outcome> > (<<Adherence indicator> > = < <Trial arm indicator>>), vce(robust)
The Zoledronate versus Ibandronate Comparative Evaluation (ZICE) trial
ivregress 2sls < <Outcome> > <<Predictors of outcome> > <<Predictors of adherence> > (<<Adherence in experimental arm> > <<Adherence in standard treatment arm> > = < <Trial arm indicator> > <<Predictors of outcome> > <<Trial arm * Predictors of outcome interactions> > <<Predictors of adherence> > <<Trial arm * Predictors of adherence interaction>>), vce(robust)
lincom[<<Experimental treatment arm effect> > - < <Standard treatment arm effect>>]
For the CODA trial, the adherence indicator was one variable that was 1 if the participant was allocated to the OD arm (experimental intervention) and adhered, 0 if they were allocated to the OD arm and did not adhere, and also 0 if they were allocated to the TDS arm (standard care).
For the ZICE trial, as distinct causal parameters were identifiable, each arm had its own variable to denote adherence. This variable was 1 if the participant was allocated to the arm and adhered, 0 if they were allocated to the arm and did not adhere, and 0 if they were allocated to the other arm.

*OD* once daily, *TDS* three times daily

## Results

### The CODA trial

**Fig. 2:**
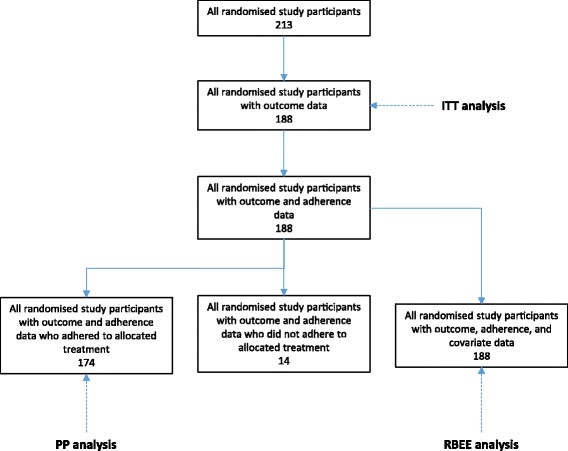
Flow diagram describing data available for each type of analysis in the Colitis Once Daily Asacol (CODA) trial

The analysis is based on 188 randomised participants with outcome data. In total, 174 participants adhered to their study medication (92.6%), with these making up the PP population (Fig. 2). The percentage of participants adhering to study medication was higher in those randomised to the intervention arm compared to the active control arm (95.7% and 89.4%, respectively).

Overall, 56 participants relapsed within the 12-month follow-up period (29.8% of all participants). The percentage of participants who relapsed was lower in the intervention arm compared to the active control arm (24.5% and 35.1%, respectively). The main trial analysis based on complete cases demonstrated that the relapse rate was 10.6 percentage points higher in those randomised to the TDS arm compared to in the OD (95% confidence interval (CI): −2.5 to 23.8 percentage points). As the lower limit of the 95% CI did not include −10%, and this was also confirmed in the PP analysis, the findings confirmed the non-inferiority of the OD regimen compared to the TDS regimen.

#### Predictors of outcome

Predictors of relapse were age (participants aged 65 years or older had decreased odds of relapsing during the follow-up period), length of remission (participants in remission for at least 12 months had decreased odds of relapsing during the follow-up period), and endoscopy findings at baseline (participants with non-normal endoscopy findings at baseline had increased odds of relapsing during the follow-up period) (Table 2).

**Table 2:** Multivariable determinants of outcome in the Colitis Once Daily Asacol (CODA) trial (odds of relapsing during the 12-month follow-up period)

Variable	Adjusted odds ratio	95% Confidence interval		*p* value
		Lower	Upper	
Age at baseline (≥65 compared to <65 years)	0.30	0.10	0.88	0.028
Length of remission (≥12 compared to <12 months)	0.34	0.14	0.81	0.014
Endoscopy findings at baseline (non-normal compared to normal)	4.14	2.04	8.39	<0.001

#### Predictors of adherence

When conditioning on the above variables, smoking status at baseline was the only variable that remained independently associated with participants adhering to their study medication at the 10% significance level (Table 3). Compared to non-smokers, the odds of participants adhering to their study medication was higher in those who were ex-smokers. However, smoking status did not differentially predict adherence across the two arms (i.e. the interaction between smoking status and trial arm was not statistically significant).

**Table 3:** Multivariable determinants of adhering to medication in the Colitis Once Daily Asacol (CODA) trial

Purpose	Variable	Adjusted odds ratio	95% Confidence interval		*p* value
			Lower	Upper	
Associated with disease status at 12 months (relapsed/still in remission)	Intervention (OD arm compared to TDS arm)	2.61	0.75	9.03	0.131
	Age at baseline (≥65 years compared to <65 years)	2.42	0.27	21.70	0.430
	Length of remission (≥12 months compared to <12 months)	1.05	0.29	3.75	0.940
	Endoscopy findings at baseline (non-normal compared to normal)	0.31	0.10	1.01	0.053
Associated with adherence to study medication	Smoking status at baseline (current smoker compared to non-smoker)	1.31	0.25	6.79	0.076
	Smoking status at baseline (ex-smoker compared to non-smoker)	11.46	1.40	94.01	

*OD* once daily, *TDS* three times daily

#### Structural mean model

It was not possible to derive two distinct causal parameters based on observed data, as there were no baseline variables differentially associated with adherence for each of the arms. Given that the definition of adherence was binary, the only sensible analysis was to consider the standard treatment (active control) as the ‘placebo’ group and use standard SMM methods.

**Fig. 3:**
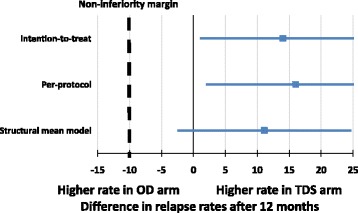
Forest plot of the difference in relapse rates in the Colitis Once Daily Asacol (CODA) trial for various analysis sets

The SMM analysis found that after adjusting for adherence, the relapse rate was 11.1 percentage points higher in those randomised to intervention. The 95% CI did not contain −10% (95% CI −2.5 to 24.7 percentage points), and non-inferiority could be confirmed based on this analysis (Fig. 3).

### The ZICE trial

**Fig. 4:**
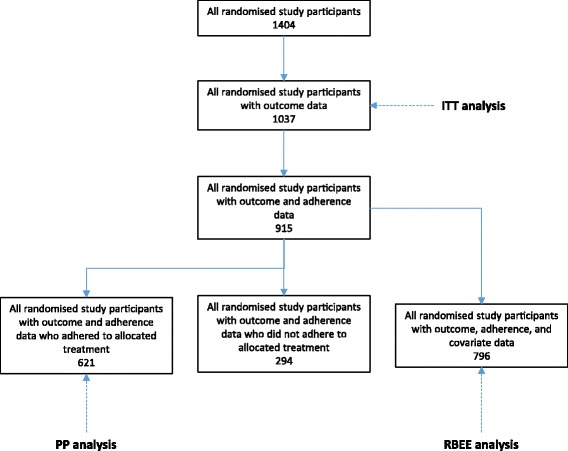
Flow diagram describing data available for each type of analysis in the Zoledronate versus Ibandronate Comparative Evaluation (ZICE) trial

The analysis is based on 1037 randomised participants with outcome data. In total, 621 of 915 participants with adherence data adhered to their study medication (67.9%), with these making up the PP population. The percentage of participants adhering to study medication was higher in those randomised to the OIA arm compared to the IZA arm (77.4% and 60.7%, respectively). Baseline covariate data were available for 796 participants. This made up the SMM population (Fig. 4).

Overall, 382 participants experienced an SRE within the 12-month follow-up period (36.8% of all participants). The percentage of participants who experienced an SRE was higher in the OIA arm compared to the IZA arm (38.3% and 35.4%, respectively). The trial analysis based on complete cases demonstrated that the SRE rate was 3.0 percentage points higher in those randomised to the OIA arm compared to in the IZA (95% confidence interval (CI) −2.9 to 8.8 percentage points) and concluded that OIA was inferior to IZA.

#### Predictors of outcome

The odds of experiencing an SRE within the first 12 months of the study were higher in participants with higher BMI scores, in participants who had poor role functioning, worse nausea/vomiting symptoms, had experienced an SRE in the 3 months prior to the study, or had recently used pain medication. The odds of experiencing an SRE within the first 12 months of the study were lower in women than in men, in participants with higher overall general health, and in participants with increasing dyspnoea (Table 4).

**Table 4:** Multivariable determinants of outcome in the Zoledronate versus Ibandronate Comparative Evaluation (ZICE) trial (odds of experiencing a skeletal-related event during the first 12 months)

Variable	Adjusted odds ratio	95% Confidence interval		*p* value
		Lower	Upper	
Gender (female compared to male)	0.23	0.06	0.88	0.032
18.5 kg/m^2^ < BMI ≤ 25 kg/m^2^ (normal/healthy weight) compared to ≤ 18.5 kg/m^2^ (underweight)	6.16	0.75	50.65	<0.001
25 kg/m^2^ < BMI ≤ 30 kg/m^2^ (overweight) compared to ≤ 18.5 kg/m^2^ (underweight)	6.85	0.84	56.13	
30 kg/m^2^ < BMI ≤ 35 kg/m^2^ (moderately obese) compared to ≤ 18.5 kg/m^2^ (underweight)	13.17	1.59	108.81	
35 kg/m^2^ < BMI ≤ 40 kg/m^2^ (severely obese) compared to ≤ 18.5 kg/m^2^ (underweight)	6.99	0.81	60.39	
BMI > 40 kg/m^2^ (very severely obese) compared to ≤ 18.5 kg/m^2^ (underweight)	13.11	1.44	119.65	
QLQ-C30 global health domain (per unit increase)	0.98	0.98	0.99	0.001
QLQ-C30 role functioning domain (per unit increase)	1.01	1.00	1.02	0.005
QLQ-C30 nausea / vomiting domain (per unit increase)	1.01	1.01	1.02	<0.001
QLQ-C30 dyspnoea domain (per unit increase)	0.99	0.99	1.00	0.056
SRE within the three months prior to baseline compared to no SRE within three months prior to baseline	1.56	1.14	2.13	0.006
Recent use of pain medication at baseline compared to no recent use of pain medication	1.63	1.08	2.46	0.019

#### Predictors of adherence

After conditioning on the above, both cognitive functioning and use of chemotherapy were independently associated with adhering to study medication differently in the two arms (Table 5). The results from the model suggest that the odds of adhering to study medication are:

**Table 5:** Multivariable determinants of adhering to medication in the Zoledronate versus Ibandronate Comparative Evaluation (ZICE) trial

Purpose	Variable	Adjusted odds ratio	95% confidence interval		*p* value
			Lower	Upper	
Associated with the development of a SRE within 12 months	Gender (female compared to male)	1.29	0.36	4.55	0.697
	18.5 kg/m^2^ < BMI ≤ 25 kg/m^2^ (normal/healthy weight) compared to ≤ 18.5 kg/m^2^ (underweight)	2.19	0.74	6.47	<0.001
	25 kg/m^2^ < BMI ≤ 30 kg/m^2^ (overweight) compared to ≤ 18.5 kg/m^2^ (underweight)	2.05	0.70	6.00	
	30 kg/m^2^ < BMI ≤ 35 kg/m^2^ (moderately obese) compared to ≤ 18.5 kg/m^2^ (underweight)	2.35	0.79	7.03	
	35 kg/m^2^ < BMI ≤ 40 kg/m^2^ (severely obese) compared to ≤ 18.5 kg/m^2^ (underweight)	3.07	0.95	9.95	
	BMI > 40 kg/m^2^ (very severely obese) compared to ≤ 18.5 kg/m^2^ (underweight)	3.90	1.06	14.31	
	QLQ-C30 global health domain (per unit increase)	1.00	1.00	1.01	0.358
	QLQ-C30 role functioning domain (per unit increase)	1.00	1.00	1.01	0.300
	QLQ-C30 nausea/vomiting domain (per unit increase)	1.01	1.01	1.02	0.000
	QLQ-C30 dyspnoea domain (per unit increase)	1.00	0.99	1.00	0.547
	SRE within the 3 months prior to baseline compared to no SRE within 3 months prior to baseline	1.07	0.79	1.46	0.660
	Recent use of pain medication at baseline compared to no recent use of pain medication	0.65	0.45	0.94	0.021
Differentially associated with adherence by trial arm	Orally administered ibandronic acid arm (main effect)	5.77	2.05	16.26	0.001
	QLQ-C30 cognitive functioning (main effect)	1.01	1.00	1.02	0.005
	Orally administered ibandronic acid arm x QLQ-C30 cognitive functioning (interaction)	0.99	0.98	1.00	0.061
	Use of chemotherapy at baseline (main effect)	2.12	1.28	3.53	0.004
	Orally administered ibandronic acid arm x Use of chemotherapy at baseline (interaction)	0.47	0.22	1.02	0.057

- Higher for participants allocated to the OIA arm, with the lowest levels of cognitive functioning, and not undergoing chemotherapy at baseline
- Higher as cognitive functioning increases for participants allocated to the IZA arm
- Lower as cognitive functioning increases for participants allocated to the OIA arm
- Higher for participants undergoing chemotherapy at baseline and allocated to the IZA arm
- Lower for participants undergoing chemotherapy at baseline and allocated to the OIA arm
- *BMI* Body Mass Index, IZA Intravenously administered zoledronic acid, *OIA* Orally administered ibandronic acid OIA, *QLQ-C30* EORTC QLQ-C30 score version 3.0, *SRE* Skeletal-related event,

#### Structural mean model

**Fig. 5:**
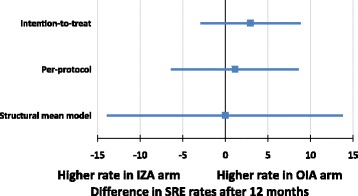
Forest plot of the difference in the proportion with skeletal-related event (SRE) in the first 12 months in the Zoledronate versus Ibandronate Comparative Evaluation (ZICE) trial for various analysis sets

Distinct causal parameters could be estimated using the ZICE data, and therefore the difference between the two arms could be calculated. After adjusting for treatment adherence, the proportion with SRE in the first 12 months was no different in either of the arms (difference in proportions 0.0, 95% CI −13.9 to 13.8 percentage points). While the point estimate from the SMM was closer to no difference, the width of the confidence interval contains any non-inferiority margin that could be justified (Fig. 5).

## Discussion

### Summary of paper

This paper investigated the use of randomisation-based efficacy estimators in non-inferiority trials. Structural mean models were fitted using a method proposed by Fischer et al., where baseline variables that predicted adherence differentially were sought to derive causal estimators in each treatment arm. This method was applied to two datasets from clinical trials involving patients in remission with ulcerative colitis (CODA) and breast cancer with bone metastases (ZICE) using standard statistical software. In the CODA trial, it was not possible to derive distinct estimators, and standard SMM methods were applied instead, treating the active control arm in the same way that a placebo arm would be treated. This analysis was consistent with the ITT and PP findings (i.e. there was evidence to suggest that OD was not inferior to TDS in terms of preventing relapse). In the ZICE trial it was possible to derive distinct estimators, and when comparing the arms the point estimate implied no difference in SRE rates between the arms, but the confidence intervals were considerably wider than the ITT and PP analyses.

### Strengths and weaknesses of the approach

To our knowledge, this is the first paper to demonstrate the potential use of randomisation-based efficacy estimators as a primary analysis in non-inferiority trials. Data from two non-inferiority trials were used, and the strengths and limitations of RBEEs and SMMs using the method proposed by Fischer et al. when applied to real-world data were established.

Both studies captured adherence to treatment differently. In the CODA trial, adherence was captured using tablet counts and in the ZICE trial adherence was captured using self-report and hospital attendance data. These methods have been demonstrated to over-estimate adherence in certain circumstances, [19–21] but they are methods that are cheap and easy to apply in large-scale randomised controlled trials, so are likely to reflect the type of data obtained in other settings (as opposed to more direct methods or electronic monitoring).

The ZICE trial used a simplified version of the original primary outcome in order to illustrate the use of these methods. One consequence of this is that while a non-inferiority margin was defined for the original primary outcome, one was not defined for the simplified version. While this could have limited the interpretation of this analysis, the confidence intervals were too wide for any NI margin to be justified, even post hoc (given that the original trial analysis suggested inferiority, this was a simplified outcome that would have had lower power than a recurrent event outcome, and the confidence interval of the SMM analysis was over twice as wide as the ITT and PP analyses).

Both studies took adherence as a quantitative measure and dichotomised it. While this was necessary for defining the analysis set, it was an approach that meant a loss of information with regards to the extent to which participants adhered to treatment. Using a binary definition of adherence (≥75%/<75% for the CODA trial and full versus not full for the ZICE trial) meant that the exclusion restriction was less likely to be plausible [14]. However, choosing an arbitrary lower threshold would have yielded estimates that were difficult to interpret, and treating adherence as a quantitative measure would have meant the additional assumption of a linear relationship between treatment adherence and treatment effect [22].

Participants with missing outcome or adherence data may have induced some selection bias in the findings presented. However, adjustments for missing data (e.g. with multiple imputation) tend to be used as secondary/sensitivity analysis in trials [23], and the purpose of this paper was to demonstrate the use of RBEEs as the main analysis in NI trials. An assessment of the impact of missing data on the interpretation of the SMM analysis can be seen in Additional file 1. Additionally, other variables that were not recorded in sufficient detail that may have influenced adherence to trial treatments, clinical outcomes, and/or dropout include the use of rescue medication and other medication that was added to a patient’s treatment plan part way through the study.

It was also decided to present an approach that could be adopted more readily, hence the use of modified least squares (MLS) for a binary outcome, rather than deriving estimates using a generalised method of moments approach [24].

### Comparisons to existing trials literature

A recently published paper investigating the comparative efficacy of two different antidepressants was the first to demonstrate the practical implementation of the SMM approach as outlined by Fischer et al. [25]. However, this approach is particularly appropriate for non-inferiority trials (as indicated in the abovementioned paper), and thus our publication complements this work by implementing this SMM approach in two non-inferiority trials. One other study has reportedly implemented this approach on a non-inferiority trial [26]. However, as this was a placebo-controlled trial, and the paper detail of the approach was lacking, it was unclear whether they applied standard SMM methodology or the extended work described by Fischer et al. Therefore, to our knowledge, this is the first publication to demonstrate how this approach works in practice for non-inferiority trials with two active interventions.

### Implications for researchers

Structural mean models could replace traditional efficacy analyses that are often reported alongside an ITT analysis in non-inferiority trials. However, this paper highlights the increase in variance experienced when fitting these models, something that can only be reduced when the models include strong predictors of adherence and outcome. Use of the method is more accurate in terms of reducing selection bias, but is likely to be less precise, and increases the importance of collecting relevant and complete baseline variables. To do this, the research team must have a good understanding of the predictors of outcome, and also the barriers/facilitators to adhering to the randomised treatments. Studies with feasibility/pilot stages could explore these aspects, as well as how best to capture this data, before progressing onto more definitive studies. The significance thresholds for inclusion of variables in this paper were higher than current practice. Future studies that collect strong baseline predictors of adherence need not use such high significance levels.

Estimating efficacy in randomised trials is valuable, as it answers a more patient-centred question than can be answered by an estimate of effectiveness. That is, “*what is the effect if I take this treatment?*”, rather than the more health care professional-centred question “*what is the effect if I offer this treatment?*” Both questions are useful, but for a patient trying to understand the effect of a treatment, the more pertinent of the two questions relates to efficacy rather than effectiveness.

By modelling the determinants of differential adherence in the different treatment arms, researchers will also gain an understanding of the circumstances under which the treatments will be better received by patients and, therefore, more likely to work. For example, in the ZICE study, we were able to demonstrate that for participants allocated to the intravenously administered zoledronic acid arm, adherence was higher for patients with higher cognitive function and for those receiving chemotherapy at baseline. Whereas for those allocated to the orally administered ibandronic acid arm adherence was lower for patients with lower cognitive function and for those receiving chemotherapy at baseline. One explanation for this could be that patients with low cognitive function could have their medicines dispensed by a care giver, which is likely to reduce forgetfulness and increase adherence. Patients receiving chemotherapy at baseline will be attending hospital regularly for these visits, and the delivery of IZA often coincided with other hospital visits for cancer therapy, thereby increasing their chances of receiving IZA treatment. The implications of this, regardless of the comparative efficacy of the treatments themselves, could be that IZA should be offered to those undergoing additional cancer treatments (or any other treatments that require regular hospital visits). OIA could be offered along with an additional intervention to increase adherence (e.g. a reminder or monitoring system), or in instances where patients were not in control of their own medication dispensing (e.g. elderly residents of nursing homes).

### Potential extensions and future work

By extending this methodology to allow for different types of outcome (e.g. binary, count, survival), this approach could be more widely used. For example, the primary analysis in the ZICE trial was based on an Anderson-Gill model (survival model with recurrent events) [27].

While not as necessary here, as a binary definition of treatment receipt is required to define an analysis set, methods of RBEEs that allow for non-linear relationships between an increase in adherence and treatment effects would be useful for capturing the complexity of some dose-response relationships more accurately.

Finally, further work is needed in order to incorporate necessary adjustments into sample size calculations for the design of trials that wish to use these methods as more than an exploratory analysis. Adjustments will likely depend on the proportion of non-adherence, as well as the number and strength of baseline predictors/instruments that are likely to be identified.

## Conclusions

In NI trials, RBEEs can provide a randomisation-respecting estimate of treatment efficacy that accounts for treatment adherence, addressing the deficiencies of both ITT and PP analysis for this study design. For NI trials involving two active treatments, RBEEs can also be modelled, remain straightforward to implement using standard statistical software, but require thorough planning during the design stage of the study to ensure that strong baseline predictors of treatment are captured.

## Additional file

Additional file 1: Data assumptions made for the ZICE trial. Descriptions of how the adherence and outcome data were derived for the ZICE study. Sensitivity analysis exploring the impact of missing data on the interpretation of the SMM analysis in the ZICE trial. (DOCX 20 kb)

## Abbreviations

BMI: Body Mass Index
CODA: Colitis Once Daily Asacol
EORTC QLQ-C30: European Organisation for Research and Treatment of Cancer 30-item Quality of Life Questionnaire developed for Cancer Patients
ITT: Intention-to-treat
IZA: Intravenously administered zoledronic acid
MEMS: Medication Event Monitoring System
NI: Non-inferiority
OD: Once daily
OIA: Orally administered ibandronic acid
PP: Per-protocol
RBEE: Randomisation-based efficacy estimator
RCT: Randomised controlled trial
SMM: Structural Mean Model
SRE: Skeletal-related event
TDS: Three times daily
ZICE: Zoledronate versus Ibandronate Comparative Evaluation
