# Supplementary material for: The use of randomisation-based efficacy estimators in non-inferiority trials
Source: Trials. 2017 Mar 9;18:117. doi: 10.1186/s13063-017-1837-3 (PMC5343391; doi:10.1186/s13063-017-1837-3)
Supplement: Additional file 1: — Data assumptions made for the ZICE trial. Descriptions of how the adherence and outcome data were derived for the ZICE study. Sensitivity analysis exploring the impact of missing data on the interpretation of the SMM analysis in the ZICE trial. (DOCX 20 kb) [file 13063_2017_1837_MOESM1_ESM.docx]

**Additional material:**

**1.** Full description of determining medication adherence in the ZICE study

Questions about adherence to study medication were asked at three initial interim visits, and then subsequently at 12-weekly visits.

Missing visit patterns were inspected, with the view to calculate adherence levels only in those with complete visit data up until the point of an event, withdrawal, death, or the end of the first 12 months.

For participants allocated to intravenous zoledronic acid:

- Adherence to intravenous zoledronic acid was based on interim and 12-weekly visit data, as participants were required to attend to receive intravenous medication. It was assumed that participants did not adhere to study medication if they either did not attend a scheduled visit, or attended but were noted as not receiving study medication as prescribed during at least one visit.

For participants allocated to oral ibandronic acid:

- Interim visits were primarily arranged so that participants allocated to intravenous zoledronic acid could receive their medication. Participants in the oral ibandronic acid arm were also invited to attend interim visits to minimise the likelihood that an increase in clinical contact in one arm could impact on trial findings. However, as it was not necessary for participants in this arm to attend visits to receive medication, and non-attendance at one or more interim visit was high, adherence to oral ibandronic acid was based on 12-weekly visit data only. It was assumed that participants did not adhere to study medication if they were noted as not receiving study medication as prescribed during at least one visit.

Adherence data were available for 1164 participants.

**2.** Full description of determining outcome (a skeletal-related event within the first 12 months) in the ZICE study

The outcome used for the ZICE study in this paper is the occurrence of a skeletal-related event (SRE) by the end of the 12 month post-randomisation follow-up period. Based on the available data (up to the end of the trial), participants were classed as one of the following:

- Reported an SRE within the first 12 months (YES)
- Reported an SRE after the first 12 months (NO)
- Alive at the end of the follow up period, no SRE reported (NO)
- Died after the end of the 12 month follow-up period, did not report an SRE in the first 12 months (NO)
- Died before the end of the 12 month follow-up period, no SRE reported (MISSING)
- Withdrew after the end of the 12 month follow-up period, did not report an SRE in the first 12 months (NO)
- Withdrew before the end of the 12 month follow-up period, no SRE reported (MISSING)

SRE outcome data were available for 1037 participants.

**3.** Impact of missing data on the interpretation of the SMM analysis

Applying a basic imputation method meant that the predictors I had originally found were no longer statistically significant. I was therefore unable to apply the SMM method as I had originally. Another approach I took, was to restrict the ITT and PP analysis to those who also feature in the SMM analysis. However, this changes the point estimates as well as widening the confidence intervals slightly (Additional Figure 1).

**Additional Figure 1:** Impact of missing data on the interpretation of the SMM analysis

*Intention-to-treat n = 1037; Per-protocol n = 621; Structural mean model n = 796

†Analysis performed in participants who were included in the structural mean model analysis. Intention-to-treat n = 796; Per-protocol n = 536
